# Supplementary material for: Molecular alterations in the integrated diagnosis of pediatric glial and glioneuronal tumors: A single center experience
Source: PLoS One. 2022 Apr 1;17(4):e0266466. doi: 10.1371/journal.pone.0266466 (PMC8975011; doi:10.1371/journal.pone.0266466)
Supplement: Attachment — Submitted filename: Rebuttal Letter.docx [file pone.0266466.s002.docx]

Rebuttal Letter

*PLoS ONE*

Editorial office

March 3^rd^, 2022

Ref: PONE-D-21-37332

Tittle: Molecular alterations in the integrated diagnosis of pediatric glial and glioneuronal tumors: a single center experience

***-- Comments from the Editorial Office --***

When submitting your revision, we need you to address these additional requirements.

1. Please ensure that your manuscript meets PLOS ONE's style requirements, including those for file naming. The PLOS ONE style templates can be found at

https://journals.plos.org/plosone/s/file?id=wjVg/PLOSOne_formatting_sample_main_body.pdf and https://journals.plos.org/plosone/s/file?id=ba62/PLOSOne_formatting_sample_title_authors_affiliations.pdf

**Response:** We checked PLoS ONE´s style requirements and corrected these issues in the manuscript. These changes are visible with the track-changes feature in the manuscript file with enabled track-changes.

2. Thank you for stating in your Funding Statement: "This study was supported in part by a grant from the National Institute of Cancer (INC) of the National Ministry of Health (Res 83/2020) and National Research Council (CONICET) PUE 2018 nº0058. E.N.D.M, M.A.L, and M.V.P are members of the CONICET Research Career Program. C.A.M. was supported by a fellowship from INC and N.C. by a fellowship from Buenos Aires City Ministry of Health. The funders had no role in study design, data collection and analysis, decision to publish, or preparation of the manuscript."

Please provide an amended statement that declares *all* the funding or sources of support (whether external or internal to your organization) received during this study, as detailed online in our guide for authors at http://journals.plos.org/plosone/s/submit-now. Please also include the statement “There was no additional external funding received for this study.” in your updated Funding Statement.

Please include your amended Funding Statement within your cover letter. We will change the online submission form on your behalf.

**Response:** “All” funding sources were already stated in the funding statement section. The statement “There was no additional external funding received for this study” was also included in the funding statement section in the cover letter. According to the PLoS guidelines, the funding statements were removed from the Acknowledgments section. Since these statements were the only thing within the Acknowledgments, this section was removed.

3. PLOS requires an ORCID iD for the corresponding author in Editorial Manager on papers submitted after December 6th, 2016. Please ensure that you have an ORCID iD and that it is validated in Editorial Manager. To do this, go to ‘Update my Information’ (in the upper left-hand corner of the main menu), and click on the Fetch/Validate link next to the ORCID field. This will take you to the ORCID site and allow you to create a new iD or authenticate a pre-existing iD in Editorial Manager. Please see the following video for instructions on linking an ORCID iD to your Editorial Manager account: https://www.youtube.com/watch?v=_xcclfuvtxQ

**Response:** I tried to add my ORCID number in the Editorial Manager system but it was not possible since an erro message appeared stating that “An identical, Validated ORCID already exists in the database. An ORCID may be linked to only one record in the database, and so you may have registered another user record in the past and retrieved/validated your ORCID for use with that.” My ORCID number is: 0000-0003-4082-0698.

4. We note that you have included the phrase “data not shown” in your manuscript. Unfortunately, this does not meet our data sharing requirements. PLOS does not permit references to inaccessible data. We require that authors provide all relevant data within the paper, Supporting Information files, or in an acceptable, public repository. Please add a citation to support this phrase or upload the data that corresponds with these findings to a stable repository (such as Figshare or Dryad) and provide and URLs, DOIs, or accession numbers that may be used to access these data. Or, if the data are not a core part of the research being presented in your study, we ask that you remove the phrase that refers to these data.

**Response:** Since the non-shown data was not a core part of the research, we removed the part of the phrase referring to it.

5. In your Data Availability statement, you have not specified where the minimal data set underlying the results described in your manuscript can be found. PLOS defines a study's minimal data set as the underlying data used to reach the conclusions drawn in the manuscript and any additional data required to replicate the reported study findings in their entirety. All PLOS journals require that the minimal data set be made fully available. For more information about our data policy, please see http://journals.plos.org/plosone/s/data-availability.

Upon re-submitting your revised manuscript, please upload your study’s minimal underlying data set as either Supporting Information files or to a stable, public repository and include the relevant URLs, DOIs, or accession numbers within your revised cover letter. For a list of acceptable repositories, please see http://journals.plos.org/plosone/s/data-availability#loc-recommended-repositories. Any potentially identifying patient information must be fully anonymized.

Important: If there are ethical or legal restrictions to sharing your data publicly, please explain these restrictions in detail. Please see our guidelines for more information on what we consider unacceptable restrictions to publicly sharing data: http://journals.plos.org/plosone/s/data-availability#loc-unacceptable-data-access-restrictions. Note that it is not acceptable for the authors to be the sole named individuals responsible for ensuring data access.

We will update your Data Availability statement to reflect the information you provide in your cover letter.

**Response:** The Data Availability statement was added to the Cover Letter and a table with all the study’s minimal underlying data was uploaded as Supplemental Material 1. Additionaly, the following phrase was added at the end of the first paragraph in the result section: “The study’s minimal underlying data set is available in Table in S1 Table.”

6. Please include captions for your Supporting Information files at the end of your manuscript, and update any in-text citations to match accordingly. Please see our Supporting Information guidelines for more information: http://journals.plos.org/plosone/s/supporting-information.

**Response:** We included a section for the Supplemental material at the end of the manuscript, which is visible with track-changes option and added the phrase: “S1 Table. Minimal Underlying data set for LGG and HGG”. The file S1_Table was created and uploaded as Supplemental material

7. Please review your reference list to ensure that it is complete and correct. If you have cited papers that have been retracted, please include the rationale for doing so in the manuscript text, or remove these references and replace them with relevant current references. Any changes to the reference list should be mentioned in the rebuttal letter that accompanies your revised manuscript. If you need to cite a retracted article, indicate the article’s retracted status in the References list and also include a citation and full reference for the retraction notice.

**Response:** The reference was revised and it is complete and correct. No retracted articles were cited.

***-- Reviewer 1 --***

The manuscript presents data on gene mutation (PCR and sequencing), fusion and deletion (FISH) and protein expression (IHC) of BRAF and Histone H3 in gliomas and correlated with treatment outcome. Molecular markers for diagnosis and prognostication in cancers are still debated. The data may contribute further clarity to this area.

**Response:** We thank Reviewer 1 for his positive comments

Suggestions for improvement:

1. Abstract, last few lines: ‘for diagnostic centers without access to NGS…’ as well as elsewhere in manuscript: This statement may alternatively be written as: These potential diagnostic/prognostic markers may be suitable as further screening tests to reduce requirement on NGS, which is not available in all laboratories.

Abstract, line 7, “Study design: FISH, IHQ …” IHQ should be replaced with IHC here and elsewhere in the manuscript.

**Response:** According to the reviewer´s suggestions, we replaced the above mentioned sentence in the Abstract. All IHQ misspellings were corrected to IHC.

Page 6, Immunohistochemical analysis, line 9, “…performed with CC1 or CC2 solutions…”: Not clear what are the CC1 and CC2 solutions.

**Response:** CC1 and CC2 are the abbreviations for Roche´s commercial solutions for cell conditioning. Since this was the only time in the manuscript that we used this abbreviation, we opted for writing it in full.

Tables 1 and 2 were not found.

**Response:** Following the journal´s instructions, Tables 1 and 2 were uploaded as separate files and not embedded in the manuscript body. In the system-produced merged pdf file, links to tables were present at the end of the document.

This manuscript could benefit from a table contrasting the distribution in molecular changes and treatment outcome of LGG and HGG.

**Response: A Supplementary table, with two separate tabs for LGG and HGG, containing all detailed information regarding molecular marker status, follow-up periods and outcome was included to this revised submission.**

***-- Reviewer 2 --***

Reviewer #2: In this manuscript Preciado et et al, provide an approach comparing different molecular tools (FISH,IHQ, and NGS), to determine the prognostic and diagnostic value, in pediatric LGG and HGG.

Overall there approach is logistic and they provided good evidence to support the conclusions they make. They make an excellent point of the usefulness of these techniques, in places where NGS is not readily available.

**Response:** We appreciate Reviewer´s 2 comment on our work.

The image quality in figure 1 a and 1 b is not the best and higher resolution images would benefit the reader, and better support the data.

**Response:** Reviewer 2 probably is referring to the figure in the merged pdf file automatically produced by the journal´s submission system for review purposes. The separate image file has much better resolution quality than the one appearing in the pdf file. We believe that the original file meets the journal´s quality requirements.

The English is mostly grammatically correct, however the writing should be reviewed as many sentences need to be re-written.

**Response:** taking into consideration reviewer´s 2 comment, we thoroughly revised the manuscript and corrected several sentences with grammatical mistakes. These changes are visible in the active track-change version of the manuscript.
